# Cerebral involvement in systemic vasculitides: Extracts from the guideline of the German neurological society

**DOI:** 10.1186/s42466-019-0016-2

**Published:** 2019-05-14

**Authors:** Peter Berlit, Markus Krämer, for the Consensus Group, Marcel Arnold, Martin W. Baumgärtel, Peter Berlit, Thomas Gattringer, Bernhard Hellmich, Markus Krämer, Peter Lamprecht, Christof Specker, Andreas Steinbrecher

**Affiliations:** 1 Deutsche Gesellschaft für Neurologie, Reinhardtstr. 27 C, 10117 Berlin, Germany; 2 Neurologie, Alfried-Krupp-Krankenhaus Essen, Alfried-Krupp-Straße 21, 45131 Essen, Germany

**Keywords:** Cerebral vasculitis, Giant cell Arteriitis (GCA), Takayasu arteritis (TA), Polyarteritis nodosa (PAN), ANCA-associated Vasculitides (AAV), Granulomatosis with polyangiitis (GPA), Microscopic polyangiitis (MPA), Eosinophilic granulomatosis with polyangiitis (EGPA), Systemic lupus erythematosus (SLE)

## Abstract

Cerebral vasculitis is a rare disorder but plays a major role in the differential diagnosis of stroke, encephalopathy and headache. This guideline was developed in order to support clinicians in the diagnosis and treatment of cerebral manifestations of systemic vasculitides and rheumatic diseases. It is based on a medline research and was developed in a modified Delphi process and approved by the involved societies.

This article is an abridged and translated version of the guideline published in *DGNeurologie*: Berlit, P. & Krämer, M. DGNeurologie (2018) 1: 17. 10.1007/s42451-018-0001-y

## Main recommendations at a glimpse

- Giant Cell Arteriitis (GCA) is the most common primary systemic vasculitis. A thoracic aortitis can be demonstrated using PET-CT or MRI in more than 50% of the patients and is associated with the risk of aortic aneurysm and dissection.
- Immediate performance of color duplex sonography in specialized emergency consultation (Fast Track Clinic) shortens the time to diagnosis, and reduces the risk of permanent visual damage. The core finding in GCA is a circular echo-poor wall edema, the so-called halo sign.
- GC therapy for remission induction is started at an initial dose of 1 mg/kg prednisolone p. o. per day. Start of dose reduction after clinical improvement and normalization of the inflammation parameter by 10 mg every 2 weeks down to 20 mg, then by 2.5 mg every 2–4 weeks down to 10 mg, then by 1 mg per month. In GCA with visual symptoms or serious vascular complications, methylprednisolone i.v. should initially be given for 3 days in a dose of 500 to 1000 mg.
- GC-sparing therapy should be initiated at the latest when there is recurrence under GC reduction and/or a GC requirement of more than 7.6 mg after 6 months. Tocilizumab is the first approved steroid-sparing therapy of GCA.
- In the treatment of Takayasu arteritis (TA) besides prednisolone (initial 0.5–1 mg/kg) in combination with MTX 15–25 mg/week infliximab (5 mg/kg i.v. day 1, 14, 42, then every 6 weeks) can be considered.
- Polyarteritis nodosa (PAN) with and without proof of hepatitis are two different entities. PAN should primarily be treated with a combination of prednisolone (1 mg/kg BW/d) and CYC if the course is organ-threatening. In the HBV-associated form, the target should be virus elimination.
- ANCA-Associated Vasculitides (AAV) are considered three different entities based on the differences in genetic background and immunological and pathological characteristics: Granulomatosis with polyangiitis (GPA), microscopic polyangiitis (MPA) and eosinophilic granulomatosis with polyangiitis (EGPA).
- GPA is associated with proteinase 3 (PR3)-specific ANCA; MPA and – less often – EGPA with myeloperoxidase (MPO)-specific ANCA.
- With neurological involvement, therapy with GC and CYC or RTX is recommended for remission induction. After remission is achieved, MTX and AZA are considered medications of equal value for remission maintenance.
- Systemic Lupus erythematodes (SLE) is an autoimmune multiorgan disease with a complex disruption of immunoregulation, which leads to a loss of immunological self-tolerance to nuclear autoantigens and formation of autoantibodies. CNS involvement is reported in 6.4 to 93% of the cases. The American College of Rheumatology described 19 different neuropsychiatric manifestations of SLE (NPSLE). Antiphospholipid antibodies (aPL) show an association with cerebrovascular manifestations in SLE, but not with inflammatory CNS manifestations.
- The specific therapy of NPSLE depends on whether the underlying impairment is inflammatory (CNS-vasculitis) or thrombo-embolic in nature. If an inflammatory process is assumed, treatment is made with GC alone or in combination with immunosuppressives (AZA or CYC). In severe, refractory NPSLE cases, plasmapheresis, IVIG and an anti-B-cell therapy with RTX are recommended. Anticoagulation appears to be superior to antiaggregants for secondary prevention of ischemic stroke in APS.

## Definition and classification

This article is an abridged and translated version of the guideline published in *DGNeurologie*: Berlit, P. & Krämer, M. DGNeurologie (2018) 1: 17. 10.1007/s42451-018-0001-y

The majority of systemic vasculitides are immunologically-caused inflammations of the vascular wall, whereby autoantibodies, circulating immunocomplexes and cell-mediated immunoprocesses play a role in the elicitation. CNS-involvement results in headaches, multifocal symptoms and encephalopathy. Involvement of the peripheral nervous system often manifests as an asymmetric painful mononeuritis multiplex. The diagnosis is based on the clinical distribution pattern, laboratory findings and the bioptic proof of vascular wall inflammation.

An overview of the various vasculitides is found in Table 1, taking the size of the affected vessel, histological characteristics and presumed pathogenetic mechanisms into account.

**Table 1:** Classification of Systemic Vasculitides according to Vessel size [27]

Large	
– Giant cell arteriitis (GCA)	
– Takayasu-Arteriitis (TA)	
Medium	
– Polyarteriitis nodosa (PAN)	
– Kawasaki-Disease (KD)	
Small (with ANCA)	
– Granulomatous with polyangiitis (GPA)	
– Microscopic polyangiitis (MPA)	
– Eosinophilic granuomatous with polyangiitis (EGPA)	
Small (with immunocomplexes)	
– Cryoglobulinemic vasculitis (CV)	
– Behçet-Syndrome (BD)	
– Collagene vascular disease (SLE, MCTD, SS)	

Especially in small vessel vasculitides, attention must be paid to the possible occurrence as part of infections (retrovirus-associated, in chronic hepatitis with or without cryoglobulinemia and postinfectious in borreliosis, VZV or cytomegaly) [31, 44].

Angiitides also occur with lymphoproliferative diseases as paraneoplastic manifestations. It is important to consider the possibility of a medication or drug-induced vasculopathy (especially RCVS) with penicillamine, propylthiouracil, tacrolimus, amphetamine, heroin and cocaine.

Typical warning symptoms of a systemic disorder are fever, weight loss, night sweats and fatigue, especially when these symptoms occur within a rheumatic symptom complex [6]. There are several systemic symptoms which are especially suspicious for vasculitides. Among these are bloody runny nose, nasal deformation (saddle nose), red eye (episcleritis and uveitis), proteinuria, edema, oliguria and new-onset arterial hypertension as evidence of renal involvement, obstructive pulmonary symptoms such as asthma, and changes in the skin and its appendages (palpable purpura, erythema nodosum, necroses of the nail-bed groove, ulcerations, Raynaud syndrome).

## Diagnostics

When the reasonable clinical suspicion of vasculitis exists based on a combination of neurological and systemic symptoms, the diagnosis must be clarified prior to initiating immunosuppressive therapy! For this, as a rule, broad organ-specific and immunological diagnostics in cooperation with a rheumatologist are required. Laboratory tests typically reveal elevation of the acute-phase proteins (ESR, CRP), a complement consumption (in immunocomplex vasculitides), leuko-and thrombocytosis and hypochromic anemia. In addition to expanded laboratory routine with determination of complement factors and immunoglobulins, the analysis of organ-specific laboratory findings is necessary. In this, microscopic urinary sediment tests with acanthocytes or erythrocytic cylinders are strong evidence of glomerular renal involvement and can also be assessed in the course of the disease as activity markers. In combination with microprotein differentiation and quantification, the indication for renal biopsy is made, not only to confirm organ involvement, but also to determine the vasculitis. CSF analysis should include the search for oligoclonal bands and the exclusion of infections. If the basic diagnostics are compatible with vasculitis, further specific examinations should be performed depending on the clinical findings: if there is suspicion of PAN, hepatitis serology is necessary, since Hepatitis B or C is present in up to 60%. If other systemic vasculitides are suspected, the search for antibodies against the cytoplasm of neutrophilic leukocytes (ANCA) should be made, whereby there is an association between EGPA and microscopic polyangiitis (MPA) with p-ANCA (anti-MPO-ANCA) and GPA with c-ANCA (anti-PR3-ANCA).

If the clinical findings suggest a suspected diagnosis of collagenosis with or without secondary vasculitis, a search for antinuclear antibodies (ANA) should be made. This parameter is very sensitive, but hardly specific. Active collagenosis is very unlikely if the ANA titer is unremarkable. If the ANA is high-titer positive, ANA differentiation must be made taking clinical symptoms into account: if photosensitivity (butterfly erythema) and polyserositis give rise to a suspicion of systemic Lupus erythematodes (SLE), a search should be made for double strand-DNA-antibodies. If a sicca syndrome is present (dry eyes, dry mouth), Sjögren- Syndrome (SS) must be tested for by determination of Anti-Ro (SS-A) and Anti-La (SS-B)-antibodies. With corresponding skin changes, proof of Anti-SCL70 antibodies gives support to the suspected diagnosis of scleroderma; U1-RNP-antibodies support the diagnosis of a mixed connective tissue disease (MCTD; Sharp Syndrome).

## Giant cell arteriitis (GCA)

GCA is the most common primary systemic vasculitis. According to the Chapel Hill Consensus Conference Nomenclature of 2012, the terms temporal or cranial arteritis should be avoided, since the temporal artery is not always affected in GCA and, on the other hand, may be affected in other vasculitides as well, for example in GPA [9].

With a prevalence of 15–44/100,000 nearly all patients are older than 50. On average, the onset of disease is around the age of 70. GCA is more frequent in women than in men (F:M = 3:1). Histologically, there is a granulomatous panarteriitis of middle and large arteries with giant cells, lymphomononuclear infiltration and stenosing of the lumen due to intima proliferation.

Predilection sites are in the branches of the external carotid artery (temporal artery, occipital artery). In 30% of cases, the posterior ciliar arteries, and in up to 50% the aortic arch and its branches are affected. Intracranial vessels, coronaries or other organ systems are rarely affected (< 1%).

GCA is a T-cell-dependent immune process with genetic predisposition (immunogenetic association with HLA-DR4 or respectively DRB1*04 alleles); various infectious agents have been discussed as elicitors (VZV, Mycoplasma pneumoniae, parvoviruses, Chlamydia).

### Clinical picture and neurological manifestations

The main symptom in > 70% is new-onset headache of piercing-stabbing quality, frequently unilateral, usually with frontotemporal emphasis. The headaches become worse on coughing or chewing. A pathognomonic symptom is jaw claudication (30% of cases). On chewing solid foods, there is increasing or new-onset pain in the temples or a painless feeling of “blocked jaw” [32], so that eating must be interrupted. This symptom is a typical clinical sign of involvement of the branches of the ECA which supply the masseter musculature. Sensitivity of the scalp is frequently reported. Headaches are often preceded by constitutional symptoms including lack of appetite, weight loss, fever, fatigue and general malaise. These patients often seem like tumor patients. In patients with isolated aortitis, fever may be the leading clinical symptom. To be feared is blindness in one or both eyes due to involvement of the ciliar arteries (30%). The frequently irreversible blindness due to anterior ischemic opticus neuropathy (AION) may be preceded by transient visual disruptions presenting as amaurosis fugax, as scintillating scotoma or transient partial visual field deficits. Affection of the ocular musculature leads to pain of eye movements, double vision and ptosis. Other brain nerves are affected much less frequently.

In 30–70% of cases, GCA and polymyalgia rheumatica (PMR) occur in association. PMR is a vasculitic minor variant of GCA with subclinical affection of large vessels in some of the patients.

Among the diagnostic criteria of PMR are subacute pains occurring in the shoulder, pelvic and neck muscles, which are frequently symmetric, accompanied by morning stiffness and improvement in the course of the day. This is frequently accompanied by depressive mood.

The central nervous system is involved via cerebral ischemias when intracranial vessels are affected (< 2%, ICA or V3- and V4-segments). Involvement of branches of the aortic arch (40–60%) leads to side-differences in blood pressure, weakened radial pulse and claudication of the arms. A thoracic aortitis can be demonstrated using PET-CT or MRI in more than 50% of the patients and is associated with the risk of aortic aneurysm and dissection.

### Imaging procedures

Duplex sonography should be performed as soon as possible, prior to or within 4 days after starting therapy with GC. The whole length of the temporal artery and its branches should be examined with a high-resolution transducer (> 10 MHz) due to segmental involvement [19]. The core finding in GCA is a circular echo-poor wall edema, the so-called halo sign. The wall edema can be quantified by measuring the Intima-Media thickness (IMT). Normal IMT values and cut-off values respectively have meanwhile been worked out for the diagnosis of GCA [55]. The value of color-duplex sonography has recently been tested in a large multicenter prospective TABUL study on 381 patients with a clinical diagnosis of GCA. The results of the study could show that in comparison with biopsy of the temporal artery, sonography has a comparably high sensitivity (93% versus 91%) and specificity (77% vs. 81%) [35]. Two current studies could show that immediate performance of a color duplex sonography in specialized emergency consultation (Fast Track Clinic) shortened the time to diagnosis, but in particular also significantly reduced the risk of permanent visual damage [16].

**18-Fluoro-2-Desoxy-D-Glucose-PET-CT** can reveal pathologically-increased uptake in the aortic arch and the supraaortal branches. The method is therefore especially useful for proof of an extratemporal manifestation of GCA which cannot be recognized in detail using sonographic diagnostics.

**Magnetic resonance imaging** of the temporal or occipital arteries demonstrates edematous vascular wall and contrast uptake in GCA [39, 41]. The sensitivity and specificity of MRI is comparable to that of sonography. Stenoses and aneurysms can be demonstrated using MR-angiography of the aortic arch. MRI appears to be less specific than PET-CT in the assessment of inflammatory activity in large vessel vasculitis.

### Biopsy

Timing of **temporal artery biopsy** must not delay the initiation of GC therapy. The biopsy should be made within 3 days after starting GC administration, if possible; but biopsy may still be diagnostic within 6 weeks after initiation of therapy. The clinically-dominant side should be selected and a biopsy length of 1.5 cm length is desirable. Biopsy is possible with ASA 100 mg and should be performed under local anesthesia. Histologically, there are lymphocytes and macrophages, multinuclear giant cells can be found in 50% of the cases. The sensitivity of biopsy is 39% only, but the specificity is 100%.

### Therapy

#### Glucocorticoid (GC)-therapy

In initiating therapy, differentiation should be made between GCA with and without visual symptoms and vascular complications. For GCA, EULAR and the British Society of Rheumatology (BSR) recommend GC therapy in considerably higher doses than in uncomplicated PMR for remission induction: Initial dose 1 mg/kg prednisolone equivalent (maximal 60 mg) p. o. per day as single daily dose in the morning. Start of dose reduction after clinical improvement and normalization of the inflammation parameters by 10 mg every 2 weeks down to 20 mg, then by 2.5 mg every 2–4 weeks down to 10 mg, then by 1 mg per month. In GCA with visual symptoms or serious vascular complications, methylprednisolone i.v. should initially be given for 3 days in a dose of 500 to 1000 mg. If there is recurrence, increase to the last effective dose plus 10 mg prednisolone is required.

#### GC-sparing therapy

Although GCA was considered in the past as a vasculitis form with likely good prognosis, current data reveal a relevant risk for recurrence and long-term complications. For example, in a large monocenter cohort of 298 patients with GCA under GC monotherapy, 133 patients suffered 2 or more recurrences within 5 years, another 80 patients suffered one recurrence [34]. Only 78 of the 298 patients (26%) suffered no recurrence. In a cohort observed for a mean 7.8 years, there was recurrence during the course in 64% of the 106 patients [3]. Patients suffering recurrence had a significantly longer duration of therapy and higher cumulative GC dose, as well as a high incidence of osteoporosis (65 vs. 32%) over the course. While mortality with an isolated temporal arteritis does not appear to be elevated, it is up to 5 times as high when the aorta is affected [30]. Data from a prospective study on 40 patients, in whom a second temporal biopsy was taken randomly after 3, 6, 9 or 12 months [38] have shown, that under a clinically effective GC monotherapy, an active arteritis could still be histopathologically diagnosed in the biopsies in 7/10 after 3 months, 9/12 after 6 months, 4/9 after 9 months and 4/9 after 12 months.

Studies in recent years also showed that two cytokine axes are relevant in the pathogenesis of RCA: the IL-6/IL-17-axis and the IL-12-Interferone-γ (INF-γ)-axis. While the IL-12/IL-17-axis can be very well suppressed by GC, the IL-12/IFN-γ-axis cannot be relevantly influenced by GC [63]. The most recent data on outcome and pathogenesis of large vessel vasculitides permit the conclusion that the majority of patients with GC monotherapy are not adequately therapied long-term and thus there is a need for more effective and more targeted therapeutic procedures. GC-sparing therapy should be initiated at the latest when there is recurrence under GC reduction and/or a GC requirement of more than 7.6 mg after 6 months.

#### Tocilizumab (TOC)

Tocilizumab is the first approved therapy of GCA [61]. Under subcutaneous TOC (162 mg) administered weekly significantly more patients were GC-free at week 52 after 26-week tapered GC than without TOC. Moreover, the GC dose with TOC could be reduced to 1862 mg compared to 3296 mg in 26-week administration and 3818 mg in 52-week administration. Prior to therapy with TOC, latent tuberculosis and viral hepatitis should be ruled out. Serious infections and previous diverticulitis are contraindications for therapy with TOC.

#### Methotrexate (MTX)

A metaanalysis of three randomized studies showed that MTX also decreases the risk of recurrence and enables GC saving [37]. Due to the high rate of GC-associated side effects in GCA, the EULAR recommended the administration of MTX as additive to GC (up to 0.3 mg/kg parenteral once a week plus 10 mg folic acid 24 h after MTX), as long as kidney and liver function are normal. This recommendation was published in 2009, so that it does not yet reflect the results of the GiACTA study and the approval of TOC. Due to considerable differences in the study protocols and based on other methodical problems (by today’s standards, very low MTX doses in the cited studies), even an indirect comparison of the efficacy and safety of MTX and TOC in the therapy of RCA is not possible. MTX is not formally approved in the therapy of RCA (therapy is thus off-label).

#### Supportive measures

Retrospective cohort studies demonstrate that the administration of ASA 100 mg daily can reduce the risk of cardiovascular events in GCA. Moreover, we recommend osteoporosis prophylaxis as cited in the current recommendations of the DVO. In analogy to the recommendations in ANCA-associated vasculitides, pneumocystitis-carinii prophylaxis with trimethoprim + sulfamethoxazole (400 + 80 mg Mon., Wed., Fri. 2 tablets each) can be considered in GC doses above 20 mg.

## Takayasu arteritis

Takayasu Arteritis (TA) refers to granulomatous aortitis with arteritis of the large vessels branching from the aortic arch (subclavian artery both sides, brachiocephalic trunc and common carotid artery), the pulmonary arteries and the coronary vessels. Unlike GCA, TA manifests mostly between the ages of 10 and 20. By definition, the first symptoms occur prior to the age of 50. Women are more frequently affected (F:M = 8:2). There appears to be a genetic predisposition [12] – at least in Asian patients associated with HLA B*52. Involvement of the subclavian arteries on both sides leads to impalpable radial pulses (pulseless disease) and to unmeasurable blood pressure in the upper extremities. The arterial hypertension which usually is present at the same time can be recognized indirectly in the left-heart enlargement. Involvement of pulmonary arteries can lead to severe pulmonary hypertension. In childhood, arterial hypertension may be the only symptom of the disease. While Takayasu Arteriitis is one of the most frequent causes of renovascular hypertension in Asian countries, the disease is rare here (1–3 new cases per 1 million inhabitants) [46]. Involvement of the CCA and the VA with stenosis or dissection can lead to numerous neurological symptoms, such as headache, blurred vision, TIA, stroke or epilepsy.

### ACR-criteria for the diagnosis of Takayasu arteritis

- **[** Patient younger than 50 years of age at the first manifestation
- **[** Claudication of the extremities
- **[** Reduced brachial arterial pulse
- **[** Blood pressure difference > 10 mmHg between the two arms
- **[** Noise over the subclavian artery or aorta
- **[** Remarkable findings in the arteriography

With 3 of these 6 criteria: Sensitivity 90.5%, Specificity 97.8%.

### EULAR-criteria for the diagnosis of Takayasu arteritis in childhood

Angiographic changes in the aorta and its branches plus one of the following findings:

- **[** Claudication of the extremities
- **[** Reduced peripheral arterial pulse
- **[** Blood pressure difference > 10 mmHg between the two arms
- **[** Noise over the aorta or its branches
- **[** Hypertension

### Diagnostics

MRI is valuable in the assessment of wall composition of the aorta, its apertures and other large arteries. Using inversion-recovery gradient echo sequences after gadolinium administration enables proof of delayed enhancement in the aortic wall. Among the typical inflammation parameters are accelerated ESR and elevated CRP. In TA, heat-shock proteins at the beginning of the cascade and Th17-cells on the end segment play a role in pathogenesis [5]. The extent of arteritis can be visualized using PET or PET-CT.

The diagnosis of TA can be made without histological confirmation. In elderly patients, however, a corresponding image can also occur in advanced atherosclerosis and as part of GCA of the large vessels.

### Therapy

There are no randomized clinical studies on the therapy of TA. Prednisolone (initial 0.5–1 mg/kg) in combination with MTX 15–25 mg/week is used for remission induction [42, 43]. The prednisolone dose is tapered in analogy to GCA. Alternatives are AZA and, especially in refractory course and critical ischemia in the extremities, the combination of prednisolone with bolus-CYC 500 mg/m2 body surface (BS) i.v. This is administered at 3 to 4-week intervals over 6 to 9 cycles. Administration of the monoclonal antibody infliximab (5 mg/kg i.v. day 1, 14, 42, then every 6 weeks) can also be considered [18]. In Asia, minocycline and doxycycline as MMP inhibitors have been administered in combination with prednisolone [39]. According to recent data, TOC is promising and led in individual series to remission in 3 of 4 cases [45]. Low-molecular heparin and/or ASS are recommended as supportive measures, especially in ischemic complications. Endovascular or surgical bypass procedures may become necessary in the course: the results of revascularization procedures vary with respect to the restenosis rates. No general recommendations can be made.

## Polyarteriitis nodosa (PAN)

This necrotising systemic vasculitis of the small and middle-sized arteries leads to thrombosing and the formation of microaneurysms in the visceral vessels (not of the aorta), the vessels of the musculature and peripheral nerves. In a relevant proportion of cases, there is an association with hepatitis-B- or – much less often – -C infection. The annual incidence is cited with 1.6 cases per million, median age at onset is 46 years [15]. The idiopathic or formerly called “classic” PAN must be differentiated from the HBV-associated form which occurs considerably more often in endemic HBV regions (Eskimo populations in Alaska). PAN is an immunocomplex vasculitis, whereby other causative agents in addition to HBV may be the basis for the immunocomplex formation (HCV, HIV, Parvovirus B 19). PAN should only be diagnosed based on the ACR-criteria when an EGPA, a GPA and an MPA have been ruled out [47]. According to the EULAR criteria, PAN with and without proof of hepatitis are two different entities: the proof of HBV increases the probability of a multiplex neuritis and worsens the prognosis. The frequency of PAN appears be on the decrease thanks to better treatment of the hepatitides.

### Clinic

PAN is characterized by weight loss, myalgias, fatigue and elevated temperatures. In about 50–70% of cases, there is subacute painful multiplex polyneuritis, in 50% there is muscle and skin involvement (livedo racemosa and subcutaneous nodules). Nearly half of all patients develop vascular renal involvement with infarctions, aneurysms and stenoses with resultant renovascular arterial hypertension. Also relevant for the prognosis is the involvement of the gastrointestinal tract. In 20% of the cases, there is involvement of the CNS. Among the CNS symptoms are the variable combination of headache (34%), retinopathy (32%) and encephalopathy (23%). Focal symptoms like hemipareses, epilepsies and cranial nerve palsies are each up to 10% less frequent. Rarely, there is also spinal involvement. The prognosis depends greatly on the organ manifestations (Five Factor Score – FFS) [1]. The 5-year survival probability falls from 90% with an FFS of 0 to 65% with an FFS of > 2 [17].

### Five factor score of PAN and the EGPA

- **[** Proteinuria > 1 g/day
- **[** Serum-creatinine > 1.58 mg/dl
- **[** Gastrointestinal involvement
- **[** CNS involvement
- **[** Cardiac involvement

### Diagnostics

Laboratory tests reveal elevated ESR-and CRP with thrombocytosis and anemia, occasionally a complement consumption (C3 and C4 low) and circulating immunocomplexes. HBV-serology is positive in 40%, a hepatitis C or an HIV-infection is less frequently the underlying cause. The angiographic picture of vasculitis with aneurysms and segmental stenoses and vascular occlusions is found in the vessels supplying the kidneys, mesenterium and brain. Angio-MRT or Angio-CT examinations are less invasive and can be helpful as alternatives. Angiographic findings can support the diagnosis, but they should be histologically confirmed (kidneys, skin, nerve-muscle).

### ACR-criteria for the classification of polyarteriitis nodosa (PAN)

- **[** Weight loss > 4 kg since disease onset
- **[** Livedo racemosa
- **[** Unexplained pain or swelling of the testes
- **[** Myalgia, feeling of heaviness in the legs
- **[** Mononeuritis or polyneuropathy
- **[** Diastolic blood pressure elevation > 90 mmHg
- **[** Serum-creatinine > 1.5 mg/dl
- **[** Proof of hepatitis virus in serum
- **[** Pathological arteriogram (aneurysms, occlusions)
- **[** Typical histology

With 3 of these 10 criteria: sensitivity 82.2%, specificity 86.6%, if other causes for the clinical picture have been ruled out [51].

### Therapy

Therapy of PAN depends on the severity and etiology [42, 43]. In the HBV-associated form (HBV-PAN), the target should be virus elimination. In serious cases, plasma exchange treatment and the use of immunosuppressives may become necessary.

PAN should primarily be treated with a combination of prednisolone (1 mg/kg BW/d) and CYC if the course is organ-threatening, whereby the duration is unclear. Milder courses of the disease can be treated with conventional immunosuppressives like MTX or AZA. Casuistic use of newer therapeutics like MMF, RTX, Infliximab and TOC has been reported [54, 58].

## ANCA-associated vasculitides (AAV)

Three diseases count as AAV: granulomatosis with polyangiitis (GPA), microscopic polyangiitis (MPA) and eosinophilic granulomatosis with polyangiitis (EGPA). AAV are necrotizing systemic vasculitides affecting mostly the small intraparenchymal vessels (small arteries, arterioles, capillaries, venules) and less frequently middle-size vessels. They are termed pauciimmune vasculitides due to the lack of or low immunocomplex deposits [26]. GPA is associated with proteinase 3 (PR3)-specific ANCA; MPA and – less often – EGPA with myeloperoxidase (MPO)-specific ANCA.

AAV are considered three different entities based on the differences in genetic background and various independent immunological and pathological characteristics, like extravascular necrotizing granulomatosis in GPA and the eosinophilic tissue infiltration in EGPA.

AAV are the second most frequent vasculitis group after GCA. An incidence of 10–20 per million inhabitants per year and a prevalence of 120–140 per million inhabitants have been reported for AAV in Europe and the USA. GPA is the most frequent AAV in Europe and the USA. By contrast, MPA is the most frequent AAV in Japan and China. These differences in the frequency of GPA and MPA in various populations indicate the importance of the genetic background and of environmental factors in the immunopathogenesis of AAV. Phenotypically, localized AAV is differentiated from early-systemic and generalized AAV. Generalized AAV is the most frequent phenotype (85–90% of the cases). Early-systemic or localized courses are less frequent (10–15% of the cases). Only 10% of the localized GPA cases convert in the course of the disease to generalized GPA, i.e. as a rule, the original phenotype remains even in recurrences. Moreover, courses with serious disease intensity and therapy-refractive courses have been defined within clinical studies (Table 2).

**Table 2:** European League Against Rheumatism (EULAR)-Definitions of AAV-Phenotypes (localized, early-systemic, generalized) and disease courses (serious, refractory) [42, 43]

Localized	upper and/or lower respiratory tract +/−
Early systemic	without impaired organ function or life-threatening disease +
Generalized disease	with impairment of renal or other organ function, Creatinine < 500 μmol/l +
Serious	renal or vital organ failure, Creatinine ≥500 μmol/l +
Refractory	progressive disease which does not respond to standard therapy +

*Abbreviations*: *+* ANCA-positive, *−* ANCA-negative

### Clinical signs

As a result of the underlying systemic small vessel vasculitis, the three AAV have a number of clinical signs in common. The most frequent manifestation (80–90% of cases) is a pulmorenal syndrome with pulmonary infiltrates, dyspnea and hemoptysis due to pulmonary capillaritis and a rapid-progressive glomerulonephritis with elevation of the serum creatinine and nephritic sediment. Renal involvement is less frequently found in EGPA (ca. 30%). Renal-limited AAV are less frequent. They are usually MPO-ANCA-positive. Prodromes are lack of appetite, arthralgias, myalgias – and rhinitis and/or sinusitis in GPA and EGPA. They often precede the manifestation of a pulmorenal syndrome by weeks or months. Fulminant AAV courses are less frequent. Patients with EGPA often have a long prior history of bronchial asthma and allergic rhinitis.

The most frequent manifestations in the peripheral nervous system are polyneuropathy and multiplex-neuritis and in the central nervous system CNS vasculitis.

Isolated cases with hypertrophic pachymeningitis have been described in GPA [57]. Extravascular necrotizing granulomatosis usually affects the lower and/or upper respiratory tract in GPA, while extravascular eosinophilic tissue infiltration in EGPA may affect various organs, like the lungs, gastrointestinal tract and heart (perimyocarditis) [13, 26]. Granulomatous infiltration and vasculitis of the Orbita and base of the skull in GPA may result in exophthalmus, episcleritis, cranial nerve lesions including compression of the optic nerve as well as diabetes insipidus [29].

### Diagnostics (Table 3)

Elevation of ESR and CRP, mild leucocytosis and reactive thrombocytosis are revealed in laboratory tests. In EGPA, there is usually hypereosinophilia of > 1.500/μl. Anemia is often found which may be elicited by the inflammation, but also renal in origin or induced by toxic effects of the immunosuppressive therapy. AAV are accompanied by normal or elevated complement levels. The prognosis is poorer in a subgroup of patients with reduced C3-complement level [14]. Elevated serum-creatine and nephritic sediment are found in renal involvement.

**Table 3:** Chapel Hill Consensus Conference (CHCC) Nomenclature for Granulomatosis with Polyangiitis (GPA), Microscopic Polyangiitis (MPA) and Eosinophilic Granulomatosis with Polyangiitis (EGPA)

GPA	
Extravascular necrotizing granulomatous inflammation of the upper and/or lower respiratory tracts	
Systemic necrotizing small vessel vasculitis	
Frequent necrotizing glomerulonephritis	
MPA	
Systemic necrotizing small vessel vasculitis	
Frequent necrotizing glomerulonephritis and pulmonary capillaritis	
No granulomatous inflammation	
EGPA	
Extravasal eosinophile-rich and necrotizing granulomatous inflammation of the respiratory tract	
With bronchial asthma and eosinophilia-associated systemic necrotizing small vessel vasculitis	
eosinophilic infiltrate *(≥ 4 of 6 critera)*	

ANCA are specific markers for AAV. GPA is associated with PR3-ANCA, − MPA and less often EGPA with MPO-ANCA. In cases of known or assumed AAV, the use of a high-quality immunoassay is recommended as the primary screening method for proof of PR3- and MPO-ANCA [11]. Imaging procedures like MRI of the head for evaluation of the paranasal sinuses, Orbita and the CNS, thoracic CT and abdominal sonography, lung function and broncho-alveolar lavage (BAL), complete the clinical diagnostics. The diagnosis can be confirmed by histological examination of a biopsy of an affected organ. Biopsies can be taken from the kidney, lung (transbronchial in BAL, less frequently transthoracic methods), sural nerve, skin and musculature. Up to 3 biopsies may be necessary in the ENT area. The first ENT biopsy is diagnostic in only ca. 30–40% of the cases [29].

### Differential diagnoses

Important differential diagnoses are other chronic inflammatory systemic diseases, infectious diseases and tumors. It must be pointed out that infection-triggered ANCA may be found in some chronic infectious diseases, such as subacute bacterial endocarditis (SBE). If the SBE is associated with pulmonary infiltrates and elevation of serum-creatinine which is prerenal and/or elicited by focal nephritis, the clinical picture may imitate that of AAV. Contrary to AAV, however, SBE usually shows pronounced complement reduction!

Other diseases with pulmorenal syndrome are cryoglobulinemic vasculitis, SLE, infections (legionellosis) and medication-induced forms (phenprocoumon overdose among others). Local inflammatory destructive nasal diseases, such as cocaine-induced midline destructive lesion (CIMDL), extranodal NK/T-cell lymphoma of the nasal type and rhinocerebral mucormykosis must be delineated from localized GPA [29].

### Therapy

For therapy of AAV recommendations exist from the European League Against Rheumatism (EULAR), the European Renal Association – European Dialysis and TransplantAssociation (ERA-EDTA), the German Rheumatology Society and the EGPA Consensus Task Force [22, 56].

#### Remission induction in vital threat and/or organ dysfunction

In a life-threatening initial manifestation and/or which impairs organ function or a major recurrence, i.e. a recurrence with severe organ involvement, therapy with GC and CYC or RTX is recommended for remission induction (Table 4). In young patients, in whom preservation of fertility is of great importance, an RTX therapy should be preferred [56, 64].

**Table 4:** Dosage Details for Remission-inducing Immunosuppressive Therapy in Initial Manifestations which impair Organ Function or are Life-threatening, or in Major Recurrences of an AAV [24, 56, 64]

Prednisolone	1 mg/kg (maximal 80 mg) daily, reduction to 7.5–10 mg daily within 12 weeks
RTX	375 mg/m2 i.v. weekly for 4 weeks
CYC-Bolus Therapy	15 mg/kg (maximal 1200 mg) i.v., the first 3 boli at 14-day intervals, the following3 boli (and others as indicated) at 21-day intervals
Oral CYC Therapy	2 mg/kg (maximal 200 mg daily)

*Abbreviation*: *i.v.* Intravenous

#### Cyclophosphamide (CYC)

Intravenous bolus therapy is just as effective as oral CYC therapy for remission induction in serious AAV. It differs, however, from oral administration with respect to the higher risk of recurrence with a lower rate of side effects. The dose must be adjusted for age and renal function.

#### RTX

Two randomized-controlled Phase 3 studies (RITUXVAS and RAVE) have shown that remission induction with the Anti-CD20-Antibody RTX is not inferior in efficacy to intravenous or oral CYC-therapy in initial manifestation or recurrence of GPA and MPA [28, 60]. In recurrences, RTX-therapy was even superior to oral CYC-therapy in the RAVE Study. GC-medication was tapered off after 5 months in the RAVE study. In the RITUXVAS Study it was reduced within 6 months to 5 mg prednisolone daily, then continued. In both studies, after remission had been achieved, therapy with AZA was continued in the CYC study arm for remission maintenance. The patients in the RTX study arm received no remission-preserving therapy. The high recurrence rate of 30–40% after 2 years in both therapy arms emphasizes the necessity of remission-preserving therapy after remission induction [29, 56].

It must be taken into consideration in the decision for remission-inducing therapy that the two studies (RITUXVAS and RAVE) differed with respect to their patient collectives and therapy protocols (intravenous versus oral CYC therapy). In the RITUXVAS study, patients with severe renal involvement with an average glomerular filtration rate between 10 and 20 ml/min/1.73 m2 were included, in the RAVE study patients had good renal function. In the RITUXVAS study, depending on the severity of renal involvement, patients received an intravenous CYC infusion in addition to their first and third RTX administration. In deciding on remission-inducing therapy in severe organ involvement, therefore, it should be taken into account that the evidence for RTX therapy is based on a combination of RTX with CYC therapy and not on RTX therapy alone [56].

In case series, efficacy of remission-inducing therapy with RTX was also demonstrated in patients with initial manifestation or therapy-refractive course of EGPA [40].

#### Plasma separation

Plasma separation can be considered on an individual basis in rapid-progressive glomerulonephritis or severe alveolar hemorrhage in GPA and MPA. Despite short-term therapeutic success, no long-term benefit has been shown for plasma separation compared to immunosuppressive standard therapy. Plasma separation is not effective in EGPA [56].

#### Mepolizumab

Remission was achieved in a randomized-controlled Phase 3 study of the anti-IL-5-antibody mepolizumab (300 mg subcutaneous every 4 weeks) in about 50% of patients with a recurrence or therapy-refractive course of EGPA in a period of 52 weeks. Moreover, a steroid-sparing effect could be demonstrated and the rate of recurrence reduced [62]. Mepolizumab is approved thus far for therapy of steroid-refractive bronchial asthma in a dose of 100 mg subcutaneous every 4 weeks [48].

#### Therapy refractive course

About 10 to 15% of patients do not respond to the initially-selected remission-inducing therapy. In therapy-refractive AAV over more than 4 weeks, or in chronic persistent disease, a switch is recommended from CYC to RTX therapy or vice-versa. In addition, intravenous administration of immunoglobulins (IVIG) can be considered in case of persistent disease activity. Therapy with interferons can also be considered as second- or third-line therapy in EGPA [56].

#### Remission induction without organ dysfunction and no threat to life

Remission induction with GC and MTX, and in contraindication for MTX therapy with MMF, is recommended when there is no organ function impairment and no life-threatening manifestations.

Therapy or a non-threatening minor recurrence consists of transient increase in the corticoid dose and, if appropriate, intensification of remission-maintenance therapy [56, 64]. In EGPA, AZA is used for remission induction, or MTX if AZA is contraindicated.

#### Remission-preserving therapy

After remission is achieved, usually 3–6 months after initiation of remission induction, a switch is made to remission-maintenance therapy. MTX and AZA are considered medications of equal value for remission maintenance. Therapy with low-dose RTX can be considered in case of contraindications, intolerance or previous therapy failure of AZA or MTX in light of the positive results of a Phase 3 study on remission maintenance with RTX compared to AZA therapy [23, 56, 64]. In cases of contraindications, intolerance or previous failure of these medications, Leflunomid or MMF can alternatively be considered as therapy options (Table 5).

**Table 5:** Dosage Details as Recommended for Remission-Maintenance Immunosuppressive Therapy [42, 43, 56, 64]

AZA	2 mg/kg oral daily
MTX	20–25 mg oral or parenteral weekly
RTX	500 mg i.v. on day 0 and 14, then every 6 months
Leflunomid	20–30 mg oral daily
MMF	2 g oral daily

*Abbreviation*: *i.v.* Intravenous

Remission-maintenance therapy should be conducted for at least 2 years. Therapy de-escalation with tapered reduction of the steroid dose and dose reduction of the immunosuppressive therapy can be made after 12 months, after assessment of the individual risk and is always associated with an inherent risk of recurrence [56]. Whether remission-maintenance therapy for more than 2 years is associated with a lower recurrence risk or other factors, like the PR3-ANCA Status, have predictive value with respect to the risk of recurrence, is currently a matter of controversial discussion.

#### Supportive therapeutic measures

Immunosuppressive therapy should be closely controlled. Recommendations for the adjustment of the CYC dose according to renal GFR and the patient’s age, for antiemetic therapy, Vitamin D substitution under GC, vaccinations, preservation of fertility and family planning, uroprotection with 2-mercaptoethansulfonate-sodium (Mesna) in CYC therapy and Pneumocystis-jirovecii prophylaxis with cotrimoxazol in CYC and RTX therapy follow the national and international recommendations.

## Systemic lupus erythematodes (SLE)

### Definition, epidemiology and etiology

Systemic Lupus erythematodes (SLE) is an autoimmune multiorgan disease with high variability in presentation and course of disease. The incidence in Europe is ca. 2–4 per 100,000 person-years, its prevalence about 40/100,000 with a worldwide tendency to increase in the past decades. Women are considerably more often affected than men (4:1 to 10:1), with a disease peak around the 4th decade of life [52]. The cause is a complex disruption of immunoregulation, which probably leads to a loss of immunological self-tolerance to nuclear autoantigens and formation of autoantibodies via a defect in the clearance of apoptotic materials and then also immunocomplexes, which result in tissue damage through uncontrolled proinflammatory cytokine and cell-recruitment patterns [36].

CNS involvement – depending on the precision of its definition, especially according to direct or indirect ascription to the underlying disease and the test procedures used – is reported in 6.4 to 93% of the cases [25].

Etiologically, differentiation must especially be made between the more frequent micro- and macroangiopathies or impaired perfusion caused by an antiphospholipid syndrome and the less frequent inflammatory-neurotoxic manifestations. These are associated with immunocomplex-mediated complement activation, elevated permeability of the blood-brain barrier, intrathecal migration of autoantibodies and local production of immunocomplexes and proinflammatory cytokines, which usually leads to diffuse neurological symptoms.

### Clinical presentation

The most frequent clinical symptoms of SLE are more or less characteristic skin changes, which are subdivided into an acute form (generalized maculopapulous exanthema, butterfly erythema, leukocytoclastic vasculitis), a subacute-cutaneous (symmetric, anular, polycyclic and/or pulosquamous/psoriasiforme skin changes in light-exposed skin areas) and a chronic discoid form [33]. Arthritides (non-destructive), serositides (abacterial), hematological manifestations (anemia, leukopenia, thrombopenia) and constitutional symptoms (fever, lymphadenopathy, weight loss, fatigue) are frequent, but not SLE-specific manifestations. Kidney involvement (Lupusnephritis-LN) is most important prognostically and occurs in appr. 50% of Lupus patients [8].

The American College of Rheumatology described 19 different neuropsychiatric manifestations of SLE (Table 6) [2], whereby better attribution models enabled more precise determination of the prevalence of a neuropsychiatric SLE (NPSLE), at 6 to 12% in the first year of diagnosis and in total at 19 to 38% [25]. If exclusively epileptic seizures and psychoses are used as the basis, only 6.4% have an NPSLE; if one includes cranial nerve palsies, cerebrovascular events, cortical dysfunction, cognitive impairment, peripheral neuropathies and transverse myelitis, the figure is 29% of SLE patients. Thirtynine % are affected by slight and unspecific NPSLE symptoms like headache. Most (50–60%) NPSLE manifestations occur at the early phase of the disease, 40–50% of which in the context of overall disease activity [7].

**Table 6:** Neuropsychiatric Syndromes in SLE (according to the American College of Rheumatology [2])

CNS	
Aseptic meningitis	
Cerebrovascular diseases	
Demyelinating syndromes	
Headache (including migraine)	
Impaired movement (Chorea)	
Myelopathy	
Seizures	
Acute states of confusion	
Anxiety disorders	
Cognitive dysfunction	
Mood disorder	
Psychoses	
PNS	
Acute inflammatory polyradiculoneuritis (Guillain-Barré-Syndrome)	
Autonomous neuropathy	
Mononeuropathies (single or multiplex manifestations)	
Myasthenia gravis	
Craial nerve palsy	
Plexus neuropathies	
Polyneuropathies	

### Diffuse NPSLE manifestations

Headache and mood disorders are the most frequent neuropsychiatric complaints among SLE patients, but usually not directly attributable to SLE. A similar statement applies for cognitive dysfunctions such as impaired memory, learning or concentration. Acute states of confusion and psychoses are less frequent (ca. 1–5% of SLE cases) manifestations, which are often unaccompanied by any structural changes (e.g. in MRI). SLE patients have an elevated risk of developing a posterior reversible encephalopathy syndrome (PRES), which is characterized by headache, personality changes, blurred vision and seizures with evidence of edematous white-matter changes in MRI and is not considered a direct manifestation of NPSLE. The pathophysiology of PRES is not well understood, autoregulatory impairments of cerebral perfusion with hyperperfusion usually in the framework of chronic blood pressure increases, possibly favored by additional endothelial changes, are discussed [25].

### Focal NPSLE manifestations

About 3 to 15% of SLE-patients suffer stroke in the course of their disease. The risk of stroke is elevated, in a large analysis of hospital registries in the USA to 1.5 [25], in cohort studies up to a relative risk of 8. Thrombotic events with arteriosclerosis accelerated by the chronic disease and its therapy, inflammatory vascular processes and states of hypercoagulability within a nephrotic syndrome or an antiphospholipid syndrome (APS) play a pathogenetic role.

Seizures develop in 8–18% of SLE patients. Directly associated with Lupus they occur most frequently at the start of the disease or during an acute episode. They do not lead to an elevated mortality [7, 25].

### Diagnosis of SLE

The interdisciplanary approach is important in this disease in order to correctly classify the “mosaics” of inflammatory skin changes, musculoskeletal and general symptoms, as well as laboratory findings like anemia, leukopenia and hypergammaglobulinemia. Antinuclear antibodies (ANA) are highly sensitive for the diagnosis of SLE, since they are found in practically all cases of *systemic* Lupus. Since they also are found in various other collagenoses (in similarly high frequency), they are not specific for SLE. Specific auto-antibodies are those against double-strand (ds)-DNA and against certain ENA-antigens (like anti-Sm). Anti- Ro/SS-A antibodies are considered specific, but also occur in Sjögren-syndrome.

For complement consumption in SLE, concurrent reduction of the complement factors C3 and C4 is of more value than that of only one complement factor [33]. In the course of disease, an increase in the ds-DNA antibodies, accompanied by a decrease in the complement factors C3 and C4, is viewed as *the* serological constellation of increased disease activity or an (imminent) episode. Even though they are not conceived for making the diagnosis of SLE, the new SLE Classification Criteria from the EULAR and ACR give an overview of the typical findings (Table 7). The important thing is that ANA must be present and no other or possibly better explanation can be found for the symptoms.

**Table 7:** EULAR/ACR-Classification Criteria for SLE (2018)

Scoring points of organ manifestations	
Constitutional symptoms	
Fever	2
Skin	
Non-scarring alopecia	2
Oral ulcers	2
Subacute-cutaneous or discoid LE	4
Acute cutaneous LE	6
Arthritis	
Synovitis in ≥2 joints or pressure pain in ≥2 joints with morning stiffness ≥30 min	6
Neurology	
Delirium	2
Psychosis	3
Seizure	5
Serositis	
Pleural or Pericardial extravasation	5
Acute pericarditis	6
Hematology	
Leukopenia	3
Thrombopenia	4
Autoimmunohemolysis	4
Kidneys	
Proteinuria > 0.,5 g/24 h	4
Lupus nephritis (histol.) Type II, V	8
Lupus nephritis (histol.) Type III, IV	10
Scoring points of immunologic findings	
Antiphospholipid antibodies	
aCL > 40 GPL or aß2GPI > 40 GPL or LA +	2
Complement	
C3 or C4 reduced	3
C3 and C4 reduced	4
Specific auto-antibodies	
a-ds-DNS-Ab	6
a-Sm-Ab	6

Prerequisite: ANA (HEp2-IFT) ≥ 1:80.  Classification as SLE starting at 10 points.Only the highest score within each domain is used for the total score. A criterion is not counted if another, more likely cause is present (such as for example infection, NPL, medications or other diseases). Criteria must not be concurrently present, a criterion is met if it was present in the documentation on one occasion, at least one criterion must be currently present

Certain autoantibodies have repeatedly been cited in connection with an NPSLE, but the practical importance is very limited [10]. A long-discussed association of antibodies against phosphorylated ribosomal proteins (anti-ribosomal P-Ak or just anti-P) with NPSLE can only be confirmed in some of the patients, and also in various other organ manifestations of SLE, so that the determination is not helpful in the individual case. It is also not clear which antibodies are found possibly only or mostly in the CSF, or whether this is only an expression of an inflammation-related disruption in the blood-brain barrier. Anti-glutamate-receptor antibodies (anti-NMDA) have been reported in 30–40% of SLE patients and appear to represent a subgroup of ds-DNA-antibodies which cross-react with NMDA-receptors in the brain. They have been found both in serum and CSF, but a clear association with NPSLE could not be demonstrated [21].

Aquaporin-4-IgG-antibodies are highly- specific (85–99%) for Neuromyelitis-optica-Spectrum Disease (NMOSD), which may occur in association with SLE [25]. Since NMOSD also occurs in other autoimmune diseases (Sjögren, Hashimoto), this is probably a matter of comorbidities.

Antiphospholipid antibodies (aPL) show an association with cerebrovascular manifestations in SLE, but not with inflammatory CNS manifestations. The aPL are not autoantibodies which are specific for NPSLE. They are found in ca. 30–35% of SLE patients but are confirmatory for the presence of an antiphospholipid syndrome only starting at a certain level (40 I.E./ml and higher for Anti-Cardiolipin-Ab and Ab against β2-Glykoprotein 1) or if lupus anticoagulans (LA) are proven. An LA can often be recognized in routine diagnostics by a prolonged APPT without anticoagulation with heparin, vitamin K antagonists or direct oral anticoagulants. For the diagnosis of an APS, in addition to the conspicuous serological findings, clinical evidence of venous and arterial thromboses or repeated miscarriage are necessary. Such an APS occurs in ca. 25% of all SLE cases (that is, markedly less often than only phospholipid antibodies). Transitory ischemic attacks, territorial insults or a generalized inner and outer cerebral atrophy based on a multiinfarction syndrome occur. Aseptic cardiac valve deposits (Libman-Sacks-Endocarditis) are a potential source of cerebral embolisms in SLE with APS. An important risk constellation for thrombembolic, and also cerebral manifestations in APS is the presence of a so-called Triple-Positivity [49], i.e. concurrent proof of (highly positive) antibodies against Cardiolipin, ß2-Glycoprotein 1 and a Lupus anticoagulant in the coagulation diagnostics.

### Diagnostics of NPSLE

Diagnostic processing and differential diagnosis of neuropsychiatric symptoms in patients with SLE do not differ from those in patients without SLE who have the same neurological symptoms, and include especially thorough anamneses with respect to medications, comorbidities and the search for possible evidence of infectiological causes (e.g. promoted by immunosuppression). Since certain, more likely “vasculitic” related forms of NPSLE are associated with the overall disease activity of SLE (see above), it is important to assess them.

Laboratory tests should include a complete blood count (anemia, leukopenia, lymphopenia, thrombopenia), complete clinical-chemical parameters (kidney failure, myositis, hemolysis), urine tests (nephritic sediment), determination of CRP (infection), serum electrophoresis, respectively immunoglobulins (hypergammaglobulinemia, IgG-elevation) and parameters of plasmatic coagulation (prolonged PTT). Among specific tests, determination of ANA, C3, C4 and the antibodies against ds-DNA, cardiolipin and β2-Glycoprotein, as well as lupus anticoagulans are to be recommended [7, 25].

CSF examinations serve primariliy to rule out infections and possibly primary inflammatory CNS diseases, but they can also give evidence of an NPSLE (pleocytosis, elevated albumin, IgG-Index). Imaging procedures, electrophysiological and neuropsychological tests may help to decide whether a particular clinical manifestation is due to an NPSLE. The sensitivity of MRI in active NPSLE is, on average, 57% (64% in severe, 30% in mild symptoms of NPSLE, 76% in focal, 51% in diffuse symptoms of NPSLE), whereby the most frequent pathological pattern with focal hyperintense T2-weighted lesions in the subcortical and periventricular white matter is often to be observed in patients without active NPSLE [7, 21, 25].

### Therapy

The difficult diagnosis and differentiation are not the least important reason why – unlike Lupus nephritis – there are no randomized studies and only one prospective controlled study of NPSLE. Reliable biomarkers for the disease activity are as little available as outcome parameters for CNS involvement [21].

The EULAR recommendations for the management of NPSLE (Bertsias, Tektonidou et al. 2012) are based like the consensus report of German-speaking Rheumatologists [4] almost exclusively on Expert Consensus. The specific therapy of NPSLE depends on whether the underlying impairment is inflammatory (CNS-vasculitis) or thrombo-embolic in nature, but this is not always easy to distinguish. The assumption of both mechanisms at the same time (for example in so-called catastrophic antiphospholipid syndrome or in a hemolytic-uremic syndrome within the SLE) is rare. If an inflammatory process is assumed in NPSLE (e.g. aseptic meningitis, optic neuritis, transverse myelitis, peripheral neuropathy, refractory seizures, psychoses, acute state of confusion) and there is general disease activity, treatment is made with GC alone or in combination with immunosuppressives (AZA or CYC). The GC dose (in severe cases initially high-dose parenteral) and the aggressiveness of the immunosuppression (CYC is considerably more effective, but also more toxic than AZA) depends on the severity of the NPSLE (or other disease activity). In severe, refractory NPSLE cases, plasmapheresis, IVIG and an anti-B-cell therapy with RTX, among others, are recommended [4, 7, 21, 25].

Thrombocyte aggregation inhibition and/or anticoagulation is recommended for NPSLE manifestations of an antiphospholipid syndrome (APS) – without immunosuppression (as long as other SLE manifestations do not require it). Anticoagulation appears to be superior to aggregation inhibition for secondary prevention of arterial events (including stroke, TIA) in APS. Coagulation-active medications have also been used in ischemic optic neuropathy, chorea and in myelitis when aPL were present and immunosuppression ineffective.

## Sjögren syndrome (SS)

The disease may occur as primary SS or as secondary SS in association with rheumatoid arthritis or other collagenoses. Women are more frequently affected (F:M = 9:1).

### Clinic

The main symptoms are the sicca-symptomatics in the eye (xerophthalmia) and the mouth (xerostomia). Neurological symptoms occur in 30%, most often as distal symmetric, primarily sensory PNP associated with autonomous symptoms (Adie’s pupils, orthostatic dysregulation), biopsy demonstrates  perivascular or vasculitic infiltrates. Atactic sensory neuronopathy (ganglionitis) with pseudoathetosis, gait ataxia and small-fiber neuropathy are very characteristic; cranial nerves are often involved, especially the trigeminal, facial and statoacustic nerve. There is relatively often tubulointerstitial damage to the kidneys as an additional extraglandular manifestation of Sjögren-Syndrome. Pareses have been observed rarely within the framework of hypokalemia with renal tubular acidosis [59].

### Diagnostics

Keratoconjunctivitis sicca can be demonstrated in slit-lamp test with fluorescein or with the Schirmer test. Inflammatory cell infiltrates are found in fine needle biopsy from the pancreas. In addition to anti-Ro-autoantibodies (SSA – positive in 97% of cases) or anti-La-autoantibodies (SSB – in 78% of cases), proof of rheuma factors and polyclonal hypergammaglobulinemia are frequent. A secondary mixed cryoglobulinemia should not be overlooked, especially if there is evidence of palpable purpura; the transition to a lymphoproliferative disease is possible in this situation [50].

CNS symptomatics are extremely rare and differential diagnoses (NMOSD comorbid with Sjögren) should be sought [20].

The CSF may present a mild lymphomonocytic pleozytosis, elevated albumin as well as oligoclonal banding, which may disappear after steroid therapy. Unlike in multiple sclerosis, these can, however, be proven not only in the IgG, but also in IgA and especially IgM band [53].

### Therapy

Treatment is combined with GC and AZA; in secondary cryoglobulinemic vasculitis also with CYC or RTX.
